# Hemoglobinopathies, merozoite surface protein-2 gene polymorphisms, and acquisition of Epstein Barr virus among infants in Western Kenya

**DOI:** 10.1186/s12885-023-11063-2

**Published:** 2023-06-20

**Authors:** Perez K. Olewe, Shehu Shagari Awandu, Elly O. Munde, Samuel B. Anyona, Evans Raballah, Asito S. Amolo, Sidney Ogola, Erick Ndenga, Clinton O. Onyango, Rosemary Rochford, Douglas J. Perkins, Collins Ouma

**Affiliations:** 1 Department of Biomedical Sciences, School of Health Sciences, Jaramogi Oginga Odinga University of Science and Technology, Bondo, Kenya; 2 University of New Mexico-Kenyan Global Health Programs Laboratories, Kisumu and Siaya, New Mexico, Kenya; 3 Department of Clinical Medicine, Kirinyaga University, Kerugoya, Kenya; 4 Department of Medical Biochemistry, School of Medicine, Maseno University, Maseno, Kenya; 5 Department of Medical Laboratory Sciences, School of Public Health Biomedical Science and Technology, Masinde Muliro University of Science and Technology, Kakamega, Kenya; 6 Department of Biological Sciences School of Biological, Physical, Mathematics, and Actuarial Sciences, Jaramogi Oginga Odinga University of Science and Technology, Bondo, Kenya; 7 Kenya Medical Research Institute - CGHR, Kisumu, Kenya; 8 Department of Biomedical Sciences and Technology, School of Public Health and Community Development, Maseno University, Maseno, Kenya; 9 University of Colorado, Anshutz Medical Campus, Colorado, USA; 10 Center for Global Health, Internal Medicine, University of New Mexico, New Mexico, NM USA; 11 Research and Innovations, Maseno University, Kisumu-Busia Road Private Bag, Maseno, Kenya

**Keywords:** Alpha thalassemia, SCT, G6PD mutations, MSP-2, EBV

## Abstract

**Background:**

Epstein Barr virus (EBV)-associated endemic Burkitt’s Lymphoma pediatric cancer is associated with morbidity and mortality among children resident in holoendemic *Plasmodium falciparum* regions in western Kenya. *P. falciparum* exerts strong selection pressure on sickle cell trait (SCT), alpha thalassemia (-α^3.7^/αα), glucose-6-phosphate dehydrogenase (G6PD), and merozoite surface protein 2 (MSP-2) variants (FC27, 3D7) that confer reduced malarial disease severity. The current study tested the hypothesis that SCT, (-α^3.7^/αα), G6PD mutation and (MSP-2) variants (FC27, 3D7) are associated with an early age of EBV acquisition.

**Methods:**

Data on infant EBV infection status (< 6 and ≥ 6–12 months of age) was abstracted from a previous longitudinal study. Archived infant DNA (n = 81) and mothers DNA (n = 70) samples were used for genotyping hemoglobinopathies and MSP-2. The presence of MSP-2 genotypes in maternal DNA samples was used to indicate infant *in-utero* malarial exposure. Genetic variants were determined by TaqMan assays or standard PCR. Group differences were determined by Chi-square or Fisher’s analysis. Bivariate regression modeling was used to determine the relationship between the carriage of genetic variants and EBV acquisition.

**Results:**

EBV acquisition for infants < 6 months was not associated with -α^3.7^/αα (OR = 1.824, *P* = 0.354), SCT (OR = 0.897, *P* = 0.881), or G6PD [Viangchan (871G > A)/Chinese (1024 C > T) (OR = 2.614, *P =* 0.212)] and [Union (1360 C > T)/Kaiping (1388G > A) (OR = 0.321, *P* = 0.295)]. There was no relationship between EBV acquisition and *in-utero* exposure to either FC27 (OR = 0.922, *P =* 0.914) or 3D7 (OR = 0.933, *P =* 0.921). In addition, EBV acquisition in infants ≥ 6–12 months also showed no association with -α^3.7^/αα (OR = 0.681, *P* = 0.442), SCT (OR = 0.513, *P* = 0.305), G6PD [(Viangchan (871G > A)/Chinese (1024 C > T) (OR = 0.640, *P* = 0.677)], [Mahidol (487G > A)/Coimbra (592 C > T) (OR = 0.948, *P* = 0.940)], [(Union (1360 C > T)/Kaiping (1388G > A) (OR = 1.221, *P* = 0.768)], African A (OR = 0.278, *P* = 0.257)], or *in utero* exposure to either FC27 (OR = 0.780, *P* = 0.662) or 3D7 (OR = 0.549, *P* = 0.241).

**Conclusion:**

Although hemoglobinopathies (-α^3.7^/αα, SCT, and G6PD mutations) and *in-utero* exposure to MSP-2 were not associated with EBV acquisition in infants 0–12 months, novel G6PD variants were discovered in the population from western Kenya. To establish that the known and novel hemoglobinopathies, and *in utero* MSP-2 exposure do not confer susceptibility to EBV, future studies with larger sample sizes from multiple sites adopting genome-wide analysis are required.

**Supplementary Information:**

The online version contains supplementary material available at 10.1186/s12885-023-11063-2.

## Background

Endemic Burkitt’s lymphoma (eBL) is a B-cell tumor and the most common cancer amongst children from malaria-endemic regions of Sub-Saharan Africa, Asia, and South America [1–4]. Endemic BL has a greater than 90% association with Epstein Barr virus (EBV) infection [2]. Even though the distribution of EBV is ubiquitous, with 95% of the world’s population having been infected at some point in life, there are geographical disparities in the distribution of EBV-associated eBL with high incidence rates in malaria holoendemic regions of Western and coastal Kenya [5–7]. In Kenya, 48% of children born in the malaria-endemic lake belt region of Kisumu have an abnormal α-thalassemia genotype [8, 9]. Additionally, sickle cell trait (SCT) and *P. falciparum* have a similar distribution within malaria-endemic areas of sub-Saharan Africa with an estimated prevalence above 16.2% within Western and coastal regions of Kenya [9–11]. Carriers of the heterozygous state (HbAS) benefit from reduced malaria severity and mortality [12–14]. Mutation of the glucose-6-phosphate dehydrogenase (G6PD) gene is also widely prevalent, with an estimated 8% frequency across malaria-endemic countries [15]. Approximately 400 million people living in tropical and sub-tropical areas exhibit G6PD deficiency, with a high diversity of variants in different parts of the world [16]. Additionally, there is a causal relationship between exposure to malaria, the early age of EBV infection, and the development of eBL in children from malaria-endemic regions [17]. Although these heritable traits (i.e., SCT, α-thalassemia, and G6PD deficiency) are associated with a protective advantage against malaria disease severity [18], their association with early ages of EBV acquisition in children from malaria-endemic regions such as Western Kenya, compared to other parts of the world has not been fully characterized.

There are additional factors that influence malaria disease severity, including allele-specific MSP-2 antibodies that are associated with reduced severity [19]. Previous investigations found an association between chronic exposures to *P. falciparum* infection and expansion of latently EBV-infected B-cells, leading to high viral loads that predispose children to eBL [20]. Furthermore, infants exposed to malaria during pregnancy and children resident in malaria-endemic regions have an earlier age of EBV infection and reactivation due to repeated malaria infections, thereby increasing the risk for the development of eBL [21, 22]. Children in malaria-endemic regions are typically infected by EBV within 6 months of age with early infection associated with poorly controlled and persistently elevated EBV viral loads over time [22]. Research has also shown that infants in non-malaria endemic regions of Kenya acquire EBV at the mean age of 8 months and have a lower risk of eBL development [22]. Collectively, being infected with EBV at an early age is linked to the risk of developing eBL [22–24]. To address current gaps in knowledge, we examined the relationship between hemoglobinopathies, merozoite surface protein-2 gene polymorphisms, and the acquisition of EBV among infants (0–12 months) in a holoendemic *P. falciparum* transmission region of western Kenya.

## Materials and methods

### Study site

The study was conducted in Chulaimbo Sub-County hospital in Western Kenya, a high-risk region for eBL [23, 25]. The hospital is located 18 km Northwest of Kisumu City at an altitude of 1300 m above sea level (-0.39 latitude and 34.6383 longitudes). *P. falciparum* malaria is holoendemic with two relative peaks during the long (March to May) and short rains (October to December) [26]. This region also has high morbidity and mortality rates due to infectious diseases such as malaria, HIV-1, and eBL [6, 27]. The SCT prevalence is 16.2%, and about 40% of children are heterozygous for α-thalassemia [9, 28]. Perennial *P. falciparum* is responsible for ~ 97% of malarial infections, resulting in high childhood mortality rates [27].

### Study population

The details of the study population have been previously described [29]. In brief, mothers attending the antenatal clinic (ANC) at Chulaimbo Sub-County hospital between 2011 and 2015 were screened and then enrolled based on the following inclusion criteria: mother being a resident within a 10 km radius of the hospital, willingness to participate in the study, HIV negative status, uncomplicated vaginal delivery, singleton pregnancy, positive or negative peripheral malaria parasitemia, and < 30 weeks of gestation (See Supplementary File I). As described in [29], exclusion from the study was based on the mother’s HIV status and unwillingness to participate in the study where all mothers who were HIV positive and any that were not willing to participate in the study were excluded.

### Sample size determination and data collection

The sample size of 81 was based on the findings of a previously described study [29] where significant differences in anti EBV antibodies were observed in neonates drawn from two groups of mothers; one with malaria exposure during pregnancy and another without any malaria exposure during pregnancy in a sample size of 70. Taking a sample size of 81 in our study would thus have given an 80% power to detect any expected differences at an alpha = 0.05. In brief, available archived infant DNA previously extracted from whole blood samples collected at any longitudinal time point between 6, 8, 14, 18 weeks, and 6, 9, and 12 months were used to test for α-thalassemia, SCT, and G6PD genotypes. Of the 81 samples genotyped, the following were excluded during analysis: two that failed to amplify for the SCT, one sample homozygous α-/α-/, and one sample that failed to amplify for the African G6PD variant. Achieved mothers’ DNA, extracted from venous blood at delivery, was genotyped for the presence of MSP-2 alleles (FC27 and 3D7). The MSP-2 genotyping was performed using 70 mother-child pair DNA samples. An infant was considered *in utero* exposed to MSP-2 if the MSP-2 variant was detected in maternal venous blood collected at delivery. An infant was considered non-exposed if the MSP-2 variants were not detected from the mothers’ sample.

### Determination of infant EBV infection status

The mothers’ EBV viral loads were determined using real-time bi-plex Q-PCR as previously described [21, 22]. From the EBV viral loads generated, the following formula was used in the determination of infant EBV infection status: EBV copies per cell = EBV SQ/ (beta-actin SQ/2) where SQ is starting quantity and, 2 are the copies of beta-actin per cell. Excel spreadsheet was used for analysis and results were interpreted as EBV positive if the value from the calculation was ≥ 0.5. Results were considered negative if the value from the calculation was < 0.5. The data on infant EBV infection status was abstracted from the available records and stratified into those who were EBV-positive at any of the following time points 6, 8, 14, 18 weeks: henceforth characterized as EBV status < 6 months. The second category included EBV status ≥ 6 to 12 months, capturing EBV status at 6, 9, and 12 months.

### Laboratory procedures

#### Sickle-cell polymerase chain reaction genotyping test

A previously established protocol was adapted for this study [30]. Briefly, a TaqMan SNP genotyping assay was used following the manufacturer’s protocols (Life Technologies, Grand Island, NY). Amplification was performed in a Real-time PCR StepOne Plus thermocycler from Applied Biosystems® (Foster City, CA, USA) with a triplicate of control samples for genotype Hb AA, Hb AS, and Hb SS with molecular grade water was used as a negative control.

#### Alpha-thalassemia genotyping

Genotyping for the different α-thalassemia mutations was performed with optimization to the described method [31]. In brief, PCR was performed using the GeneAmp PCR system 9700 (Applied Biosystems) in a 25 µL reaction volume containing 2.5x GoTaq Buffer-MgCl_2_, (Invitrogen), 4.0 mM MgCl_2_, 0.4 mM mixture of deoxynucleotide phosphates, α-thalassemia 3.7 primers (Integrated DNA Technologies, IDT) 0.3 µM Forward primer (“5 AAG TCC ACC CCT TCC TTC CTC ACC 3”), 0.25 µM Reverse1 primer (“ 5ATG AGA GAA ATG TTC TGG CAC CTG GAC TT 3’), 0.25 µM Reverse 2 primer (“5 TCC ATC CCC TCC TCC CGC CCC TGC CTT TT 3’), 5 Units/µL of GoTaq DNA polymerase (Promega M3005), 5% dimethyl sulfoxide (DMSO), 0.75 M betaine (Sigma), and 3 µL genomic DNA. The following cycling conditions of initial heat activation at 94 °C for 15 min, with 39 amplification cycles of denaturation at 94 °C for 45 s, annealing at 67 °C for 1 min, and elongation for 2 min at 72 °C and a final extension at 72 °C for 10 min were used. Molecular-grade water was used as a negative control. The housekeeping gene, glyceraldehyde-3-phosphate dehydrogenase (GAPDH), was amplified to confirm the presence of human DNA from randomly picked study samples. PCR products were run against a 1 kb DNA ladder (Promega Madison, WI USA) on 2% agarose gel at 80 volts for 2 h. The resulting gel image was visualized using a UV-transilluminator gel reader (UVITEC Cambridge), and generated images were captured, labeled, scored, and stored. The generated base pair (bp) sizes were identified by [2213–2217 bp for (α α /α α)] normal gene, [2213–2217 bp (α α /α α) and 1963 bp (α-/α-/)] as heterozygous and [1963 bp (α-/α-/)] for homozygous mutant gene deletions.

#### Multiplex PCR amplification for glucose-6-Phosphate dehydrogenase gene mutation

The Glucose-6-Phosphate dehydrogenase genotyping was adopted and modified from a previously used protocol [32]. Multiplex PCR was optimized such that each of the 6 mutation positions of the G6PD gene was amplified in fragments ranging from 164 to 384 bp **(**Table 1**)**. The 15 µL PCR reaction concentrations consisted of 1µL of genomic DNA, 10X reaction buffer (Promega), 10 mM mixture of deoxynucleotide phosphates, 50 mM MgCl_2,_ 10 µM of each primer set, and 2 Units/µL of Go Taq DNA polymerase (Promega M3005). The housekeeping gene, glyceraldehyde-3-phosphate dehydrogenase (GAPDH), was amplified from randomly selected samples to confirm the presence of human DNA. Molecular-grade water was used as a negative control. The Gene-Amp PCR system 9700 Thermal Cycler (Applied Biosystems) machine was used for (PCR) under the following cycling conditions and optimized for each primer set: an initial denaturation step at 95 °C for 3 min, 11 cycles of 94 °C for 15 s, 62 °C for 15 s, and 72 °C for 30 s. This was then followed by 24 cycles of 94 °C for 15 s, 56 °C for 15 s, and 72 °C for 30 s and a 3-minute final extension at 72 °C. After amplification, 20 µL of the PCR product was loaded on a 1.5% agarose gel and run against a 50 bp DNA ladder (Invitrogen) at 80 volts for 1 h 30 min. The gel was visualized using a UV-transilluminator gel reader (UVITEC Cambridge), and the resulting images were scored and stored.

**Table 1:** Primer sequences for PCR amplification of the target regions in G6PD gene

	Mutations	Substitutions	Primer sequence (5’- 3’)	Size (bp)
**Group A1**	Viangchan Chinese	871 G > A 1024 C > T	CCAACTCAACACCCAAGGAGC GGCATGCCCAGTTCTGCCTTG	**280**
**Group A2**	Mahidol Coimbra	487 G > A 592 C > T	TGAATGATGCAGCTCTGATCC CCAGGTGAGGCTCCTGAGTAC	**293**
**Group B**	Union Kaiping	1360 C > T 1388 G > A	TGGCATCAGCAAGACACTCTC GGAGAGGCATGAGGTAGCTCC	**384**

G6PD mutation types and G6PD primer sequences. Target region were amplified based on basepair (bp) sizes

#### G6PD african A- genotyping assay

According to the manufacturer’s instructions, the G6PD 376T > C and 202 C > T polymorphisms were genotyped using the TaqMan® 5’ allelic discrimination Assay-By-Design high-throughput method [Assay ID: C_2228694_20 for rs1050829 and C_2228686_20 for rs1050828; Thermo Fisher Scientific, Carlsbad, CA, USA). The PCR was performed in a total reaction volume of 10 µL with the following amplification cycles: initial denaturation at 60 °C for 30 s and 95 °C for 10 min followed by 40 cycles of 95 °C for 15 s and 60 °C for 60 s and, a final extension at 60 °C for 30 s using allele-specific fluorescence on the StepOne Plus™ Real-Time PCR Systems. The StepOne Plus™ Software (Version 2.3) was used for allelic discrimination (Thermo Fisher Scientific, Carlsbad, CA, USA).

#### MSP-2 genotyping assay

To genotype the different MSP-2 variants in these samples, the polymorphic regions of the merozoite surface protein 2 (MSP-2) were amplified by a nested PCR assay using oligonucleotide primer pairs specific for *P. falciparum* MSP-2 alleles. The primary PCR in a 15 µL reaction volume contained 1 µL of genomic DNA, 7.5 µL GoTaq master mix, 0.5 µL forward primer (M2-OF sequence 5”-ATGAAGGTAATTAAAACA TTGTCTATTATA-3”), 0.5 µL reverse primer (M2-OR primer sequence 5”-CTTTGTTACCATCGGTACATTCTT-3”) and 5.5 µL PCR water. The PCR reaction involved 30 amplification cycles composed of initial denaturation at 94 °C for 2 min, a further denaturing step at 94 °C for 30 s, followed by annealing at 60 °C for 1 min, and an extension step for 1 min at 72 °C. Nested PCR for the specific alleles was done in a 15 µL reaction volume containing 7.5 µL GoTaq green master mix 2x (Promega M791A protocol), 0.25 µM of each primer pair (M2 forward /M2-reverse) and 1 µL of primary PCR product. Molecular-grade water was used as a negative control. Nested allele-specific primers M5 reverse for 3D7 and N5 reverse for FC27 alleles (MSP-2 family-specific nested PCR primers M5 rev 5 “- GCA TTG CCA GAA CTT GAA-3” and N5 rev5 “-CTG AAG AGG TAC TGG TAG A-3”) were used. The PCR reaction involved 30 amplification cycles composed of initial denaturation at 94 °C for 2 min, a denaturing step at 94 °C for 30 s, followed by annealing at 60 °C for 60 s, and an extension step for 90 s at 72 °C. The PCR product was then run on 1.5% agarose gel and visualized using a UV-transilluminator gel reader (UVITEC Cambridge). Due to the presence of different MSP-2 clones, the genotypes were scored based on bp within the range of 250-450 bp for each genotype.

### Data analysis

#### Analyses of sickle cell, G6PD, α-thalassemia genotypes, and EBV acquisition

Data were analyzed using IBM SPSS Statistics version 23. Before analysis, children in the study were stratified based on follow-up visits into < 6 months and ≥ 6–12 months. Chi-Square or Fisher’s test was used to compare proportions. A binary logistic regression model was used to determine the relationship between variants for SCT, G6PD, and α-thalassemia [normal α globin (αα/αα) 2213-2217bps fragments], [heterozygous carriers (-α^3.7^/αα) 1963bps and 2213-2217bps], and EBV acquisition. The association between MSP-2 variants and EBV acquisition was also determined using binary logistic regression modeling. There were no covariates included in the model. Statistical significance was defined as *p* ≤ 0.05.

## Results

### Demographic and clinical characteristics of study participants

The demographic and clinical characteristics of the study participants are presented in **(**Table 2**)**. The level of education, maternal age, gestational age, and marital status was captured for the mothers upon enrollment. The median age for the mothers was 22.0 years. Most of the mothers enrolled had secondary education (n = 43, 53.1%), were in their second trimester of pregnancy (n = 53, 65.4%), and were married (n = 57, 70.4%). Data on the prevalence of specific protozoal and helminthic infections in the mothers was also determined. Pathogenic protozoal infections of *Trichomonas vaginalis* 2.5% [2], *Entamoeba histolytica* 17.3% [13], and *Giardia lamblia* 7.4% [6], were the most prevalent. Among all the infant samples, (n = 21, 26.6%) had at least one malaria episode by 12 months of age.

**Table 2:** Demographic and clinical characteristics of study participants

Maternal characteristics		Summary	
Median age in years (± S. D) at enrollment		22.0	
**Education**			
Primary		25.9% (21)	
Secondary		53.1% (43)	
Polytechnic		14.8% (12)	
College and higher		6.2% (5)	
**Gestation age at enrolment**			
1st trimester (< 14 weeks)		12% (10)	
2nd trimester (14–27 weeks)		65.4% (53)	
3rd trimester (≥ 28 weeks)		22.2% (18)	
**Marital status**			
Single		29.6% (24)	
Married		70.4% (57)	
**Pathogenic protozoan and helminth infections**			
*Trichomonas vaginalis*		2.5% (2)	
*Ascaris lumbricoides*		3.7% (3)	
*Trichuris trichiura*		1.2% (1)	
*Ascaris lumbricoides*		3.7% (3)	
*Trichuris trichiura*		1.2% (1)	
*Schistosoma mansoni*		1% (2)	
**Infant characteristics**		**Summary**	
Gender	Male n = 41(%)		Female n = 40 (%)
EBV Positive at 12 months	46.7% (14)		53.3% (16)
EBV Negative at 12 months	52.9% (27)		47.1% (24)
*In utero* malaria exposed	38.1% (8)		61.9% (13)
Malaria non exposed	55.0% (33)		45.0% (27)
*P. falciparum* infected at 12months	38.1% (8)		61.9% (13)
Alpha thalassemia (-α^3.7^/αα)	50.0% (17)		48.6% (17)
Hb AS	41.2% (7)		58.8% (10)
G6PD A (376T > A)	50.9% (28)		49.1% (27)
G6PD A- (202 C > T/376T > A)	36.8% (7)		63.2% (12)

Demographic and clinical characteristics of mothers and infants whose samples were used in the study

### Distribution of alpha thalassemia, SCT, G6PD, and MSP-2 mutations based on EBV status < 6 months of age

A total of 81 (100%) infant samples were successfully genotyped for α-thalassemia. One infant had a -α^3.7^/-α^3.7^ genotype and was excluded from the analysis. There were 46 infants without the thalassemia mutation (αα/αα) of which, 37/46 (80.4%) were EBV-positive. There were 34 infants’ carriers of -α^3.7^/αα and 30/34 (88.2%) of these were EBV-positive with the difference between the two genotypic groups being comparable (*P* = 0.615). Among (n = 81) samples genotyped for SCT, two samples failed to amplify, therefore, n = 79 samples were used for the analysis. There were 62 infant carriers of HbAA, of which 52/62 (83.9%) were EBV-positive. A total of 17 infants had the HbAS genotype, of which 14/17 (82.4%) were EBV-positive. The differences between the groups were also comparable (*P* = 1.000). The EBV infection status for the 7 G6PD mutation types (n = 81) was as follows; 72 infants had the Viangchan/Chinese genotype with 62/72 (86.1%) being EBV-positive. The difference between carriers and non-carriers of the genotypes was comparable (*P* = 0.153). There were 76 infants with the Mahidol/Coimbra mutation of which 63/76 (82.9%) were EBV-positive. The difference between carriers and non-carriers of the genotype was comparable (*P* = 0.407). There were 66 infants with the Union/ Kaiping mutation, of which 54/66 (81.8%) were EBV-positive. The difference between carriers and non-carriers of the genotype was comparable across groups (*P* = 0.197). Among 80 infant samples genotyped for African A- variant, 19 were carriers of A-, of which 13/19 (68.4%) were EBV positive. There were 55 infants’ carriers of the A variant with 48/55 (87.3%) being EBV-positive and 6 carriers of the B variant of which 6/6 (100%) were EBV-positive. The distribution of the African A- alleles across the three groups was also comparable (*P* = 0.084). There were 70 mother samples genotyped for the MSP-2 genotypes as an indicator of *in-utero* exposure to malaria. There were 20 infants exposed *in utero* to the FC27 genotype of which 17/20 (85%) were EBV positive. There were 41 infants exposed *in utero* to the 3D7 genotype, of which 35/41 (85.4%) were EBV-positive. The difference between groups was comparable between the exposed and the unexposed for both the FC27 and 3D7 allelic groups (*P* = 0.914 and *P* = 0.921) respectively (Table 3).

**Table 3:** Distribution of hemoglobinopathies and MSP-2 based on EBV status < 6 months of age

		EBV status < 6 months			EBV status ≥ 6-12months		
Gene mutation type	n	EBV negative n (%)	EBV Positive n (%)	*P*-value	EBV negative n (%)	EBV Positive n (%)	*p*-value
**Thalassemia**							
αα/αα	46	9 (19.6)	37 (80.4)	^**b**^0.615	27 (58.7)	19 (41.3)	^a^0.413
-α^3.7^/αα	34	4 (11.8)	30 (88.2)		24 (70.6)	10 (29.4)	
**Sickle cell Status**							
Hb AA	62	10 (16.1)	52 (83.9)	^**b**^1.000	37 (74.0)	25 (40.3)	^**b**^0.249
Hb AS	17	3 (17.6)	14 (82.4)		13 (26.0)	4 (13.8)	
**G6PD Group A1**							
Viangchan 871G > A /Chinese 1024 C > T -Negative	9	3(30)	6 (66.7)	^**b**^0.153	5 (6.2)	4 (4.9)	^**b**^0.616
Viangchan 871G > A /Chinese 1024 C > T -Positive	72	10(14.1)	62 (86.1)		46 (56.8)	26 (32.1)	
**G6PD group A2**							
Mahidol 487G > A/Coimbra 592 C > T -Negative	5	0 (0)	5 (7.4)	^**b**^0.407	3 (3.7)	2 (2.5)	^**b**^0.442
Mahidol 487G > A/Coimbra 592 C > T -Positive	76	13 (16.9)	63 (82.9)		48 (59.3)	28 (34.6)	
**G6PD group B**							
Union 1360 C > T/Kaiping 1388G > A)-Negative	15	1(6.7)	14 (93.3)	^**b**^0.197	5 (6.2)	10 (12.3)	^a^0.494
Union 1360 C > T /Kaiping 1388G > A)-Positive	66	12(18.2)	54 (81.8)		41 (50.6)	25 (30.9)	
**G6PD African A-**							
A-	19	6 (31.6)	13 (68.4)	^a^0.084	14 (17.5)	5 (6.3)	^a^0.280
A	55	7(12.7)	48 (87.3)		31 (38.8)	24 (30.0)	
B	6	0 (0.0)	6 (100)		5 (6.3)	1 (1.3)	
**MSP-2**							
FC27 Negative	50	7 (14)	43 (86)	^**b**^0.914	31 (59.6)	19 (65.5)	^a^0.814
FC27 Positive	20	3 (15)	17 (85)		13 (25.0)	7 (24.1)	
3D7 Negative	29	4 (13.8)	25 (86.2)	^**b**^0.921	16 (30.8)	13 (44.8)	^a^0.263
3D7 Positive	41	6 (14.6)	35 (85.4)		28 (68.3)	13 (31.7)	

Data are presented as proportions n (%). Model covariates are SCT; Sickle cell trait (Hb AS), Hb AA; Sickle cell normal: Alpha thalassemia variants (αα/αα, -α^3.7^/αα), G6PD African variant: B; WT, A-; (202 C > T/376T > A), A;(376T > A). MSP-2 exposed as Positive and unexposed as Negative. *P*-values were determined using ^a^ Chi-Square or ^b^ Fishers exact test

### Distribution of alpha thalassemia, SCT, G6PD and MSP-2 mutations based on EBV status ≥ 6-12months of age

The EBV infection status ≥ 6–12 months of age was as follows: Among the 46 (56.8%) infants with the αα/αα genotype 19/46 (41.3%) were EBV-infected. Of the 34 infants’ carriers of -α^3.7^/αα, 10/34 (29.4%) were EBV-infected with a comparable difference between the two genotypic groups (*P* = 0.413). Out of the 79 infant samples analyzed for SCT genotype, there were 62 infants’ carriers of HbAA of which, 25/62 (40.3%) were EBV-infected. Of the 17 infant carriers of HbAS, 4/17 (13.8%) were EBV-infected and the difference between the two genotypic groups was comparable (*P* = 0.249). The distribution of the 7 G6PD variants, based on EBV status among the 81 infants was as follows: there were 72 infants with Viangchan 871G > A /Chinese 1024 C > T mutation type with 26/72 (32.1%) EBV-infected [carriers vs. non-carriers were comparable (*P* = 0.616)]. There were 76 infants’ carriers of the Mahidol 487G > A/Coimbra 592 C > T mutation type, of which 28/76 (34.6%) were EBV-infected with the difference between carriers and non-carriers being comparable (*P* = 0.442). There were 66 infants’ carriers of the Union 1360 C > T/ Kaiping 1388G > A mutation type with 25/66 (30.9%) EBV-infected [carriers vs. non-carriers were also comparable (*P* = 0.494)]. There were 55 infants’ carriers of the G6PD A variant of which 24/55 (30.0%) were EBV-infected. Infants’ carriers of the A- variant were 19 of which 5/19 (6.3%) were EBV-infected. Of the 6 infants with the B variant, 1/6 (1.3%) was EBV-infected and the difference between carriers of the three variants was comparable (*P* = 0.280). The MSP-2 genotyping used 70 mother samples of which 41 (50.6%) and 20 (24.7%) had the 3D7 and FC27 alleles, respectively. There were 41 infants with *in-utero* exposure to 3D7 with 13/41 (31.7%) EBV-infected, while 20 infants with *in utero* exposure to the FC27 [7/20 (24.1%) were EBV-infected]. The difference between carriers of FC27 and 3D7 and non-carriers was comparable (*P* = 0.814 and *P* = 0.263, respectively, Table 3).

### Association between carriage of hemoglobinopathy gene, MSP-2 allele, and EBV acquisition < 6 months of age

The relationship between hemoglobinopathies and EBV acquisition is presented in Table 4. There was no statistically significant association between hemoglobinopathies and EBV acquisition < 6 months. Neither the -α^3.7^/αα [OR = 1.824, (95% CI = 0.511–6.512), *P* = 0.354] nor the SCT [OR = 0.897, (95% CI = 0.217–3.708), *P* = 0.881) were associated with the acquisition of EBV. The odds of EBV infection were, however, higher in infants < 6 months compared to the ≥ 6–12 months age category. There was no association between EBV acquisition and the Viangchan 871G > A /Chinese 1024 C > T variant [OR = 2.614, (95% CI = 0.578–11.820), *P* = 0.212], Mahidol 487G > A/Coimbra592C > T variant [OR = 0.000, (95% CI = 0.000), *P* = 0.999, or Union 1360 C > T/Kaiping 1388G > A variant [OR = 0.321, (95% CI = 0.038–2.686), *P* = 0.295]. For the G6PD African variant, the regression model did not generate odds ratios and/or 95% CI, likely due to the smaller sample sizes within the individual group variants. However, a combination of B and A variants as the reference category established a significant association of A- and EBV acquisition **[**OR = 0.281, (95% CI = 0.081–0.978), *P* = 0.046]. *In utero* exposure to MSP-2 alleles (n = 70) did not show any significant associations between exposure to FC 27 [OR = 0.922, (95% CI = 0.213–3.990), *P* = 0.914] or 3D7 [OR = 0.933, (95% CI = 0.238–3.656), *P =* 0.921] and EBV acquisition (Table 4).

**Table 4:** Association between carriage of hemoglobinopathy genes and EBV acquisition

		EBV status < 6 months			EBV status ≥ 6-12months		
Gene mutation type	n	OR	95% CI	*P-*value	OR	95% CI	*P-*value
**α- thalassemia**							
αα/αα	46	**Ref**	-	-	**Ref**	-	-
-α^3.7^/αα	34	1.824	0.511–6.512	0.354	0.681	0.256–1.812	0.442
**Sickle cell Status**							
Hb AA	62	**Ref**	-	-	**Ref**	**-**	**-**
Hb AS	17	0.897	0.217–3.708	0.881	0.513	0.143–1.836	0.305
**G6PD Group A1**							
871G > A/1024C > T-Negative	10	**Ref**	-	-	**Ref**	-	-
871G > A/1024C > T-Positive	71	2.614	0.578–11.820	0.212	0.640	0.079–5.205	0.677
**G6PD group A2**							
487G > A/592C > T-Negative	4	**Ref**	-	-	**Ref**	-	-
487G > A/592C > T-Positive	77	0.000	0.000	0.999	0.948	0.240–3.755	0.940
**G6PD group B**							
1360C > T /1388G > A-Negative	15	**Ref**	-	-	**Ref**	-	-
1360C > T /1388G > A-Positive	66	0.321	0.038–2.686	0.295	1.221	0.325–4.592	0.768
**G6PD African A-**							
B	6	**Ref**	**-**	**-**	**Ref**	**-**	**-**
A-	19	xx	xx	1.000	0.000	0.000	0.235
A	55	xx	xx	0.999	0.278	0.030–2.544	0.257
B and A	61	**Ref**	-	-	**Ref**		
A-	19	0.281	0.081–0.978	**0.046**	0.223	0.153–1.549	0.487
**MSP-2**							
FC27 Negative	50	**Ref**	**-**	**-**	**Ref**	**-**	**-**
FC27 Positive	20	0.922	0.213–3.990	0.914	0.780	0.256–2.373	0.662
3D7 Negative	29	**Ref**	**-**	**-**	**Ref**	**-**	**-**
3D7 Positive	41	0.933	0.238–3.656	0.921	0.549	0.201–1.496	0.241

G6PD: [Group A1(Viangchan 871G > A /Chinese 1024 C > T), Group A2 (Mahidol 487G > A/Coimbra 592 C > T), Group B (Union 1360 C > T / Kaiping 1388G > A)]: SCT; Sickle cell trait (Hb AS), Hb AA; Sickle cell normal: Alpha thalassemia (αα/αα, -α^3.7^/αα): B; Wild type, A-; (202 C > T/376T > A), A;(376T > A). xx; small sample size. Data are presented as odds ratios. OR Odds ratio: CI; Confidence interval

### Association between carriage of hemoglobinopathy gene, MSP-2 allele, and EBV acquisition ≥ 6–12 months of age

Infants with -α^3.7^/αα were not significantly protected from EBV acquisition ≥ 6-12months [OR = 0.681, (95% CI = 0.256–1.812), *P* = 0.442]. The Hb AS genotype was not associated with EBV acquisition in the ≥ 6–12 months group [OR = 0.513, (95% CI = 0.143–1.836), *P* = 0.305]. None of the seven G6PD variants was associated with EBV acquisition nor were the Viangchan 871G > A /Chinese 1024 C > T variant [OR = 0.640, (95% CI = 0.079–5.205), *P* = 0.677], Mahidol 487G > A/Coimbra592C > T variant [OR = 0.948, (95% CI = 0.240–3.755), *P* = 0.940], or Union 1360 C > T/Kaiping 1388G > A variant [OR = 1.221, (95% CI = 0.325–4.592), *P* = 0.768]. The G6PD African A- mutation also showed no association with EBV acquisition [OR = 1.000, (95% CI = 0.000–0.000), *P* = 0.235], nor did the A variant [OR = 0.278 (95% CI = 0.030–2.544), *P* = 0.257]. An analysis of the MSP-2 genotypes and EBV acquisition demonstrated that *in utero* exposure to either of the MSP-2 alleles was not associated with EBV acquisition in FC 27 [OR = 0.780, (95% CI = 0.256–2.373), *P* = 0.662] or 3D7 [OR = 0.549, (95% CI = 0.201–1.496), *P* = 0.241]. Based on the odds ratios the probability of an EBV infection was generally higher < 6 months compared to ≥ 6–12 months.

## Discussion

Despite the long-held hypothesis that EBV infection during infancy and *P. falciparum* infection are risk factors for eBL, the causal relationship between early EBV infection and eBL development in children from malaria-endemic regions remains unknown [22, 33]. Endemic BL and malaria have a similar geographic distribution, and polymorphisms of RBCs such as sickle cell trait (SCT), α-thalassemia, and G6PD deficiency have been associated with protection against malaria in malaria-endemic regions [18, 34, 35]. For instance, in Western Kenya, there is a high prevalence rate of clinically relevant hemoglobinopathies [36, 37]. However, whether these hemoglobinopathies could also influence EBV acquisition is yet to be determined. Therefore, the current study sought to understand the significance of these hemoglobinopathies—SCT, alpha-thalassemia, and G6PD-mutations, in the early ages of EBV acquisition. This study hypothesized that certain hemoglobinopathies may protect against EBV acquisition at an early age.

There is a high prevalence of alpha thalassemia along the lake belt region of Kenya with the advantage of being protective in *P. falciparum* malaria disease severity [38]. Data on its role in viral infections is scanty, with studies pointing at susceptibility to transfusion-related hepatitis B and C viral infections in homozygous individuals [39, 40]. Similarly, in an experimental setup, a significant reduction in susceptibility of erythroid committed precursor cells to Dengue virus from -α^3.7^/αα compared to normal control cells has also been documented [41]. In a binary regression model, with the wild-type gene as a reference group, there was no association between the -α^3.7^/αα variants and susceptibility to EBV infection. The lack of an association could be attributed to the mechanistic effect of erythrocytic phagocytosis processes established in *P. falciparum* parasitized thalassemic red blood cells (RBCs) [42]. Based on this fact, we hypothesize that there are likely additional molecular mechanisms between thalassemic RBCs and EBV acquisition that was beyond the scope of the current study.

The effect of SCT on malaria disease has also been studied in Western Kenya revealing a 16.2% prevalence in the child population from this malaria-endemic region [9, 11]. The clinical importance of SCT in viral diseases has also been demonstrated in HIV where SCT has a protective effect in reducing the progression of HIV-1 to AIDS, and consequently a reduced prevalence of HIV-1 in the African American population [43, 44]. A combined synergistic effect between SCT and blood group O in reducing the risk of eBL in Nigeria has also been reported [45]. Nevertheless, the current study revealed that children with SCT were not significantly protected from the acquisition of EBV contrary to the previously established protective association of SCT in HIV-1 infection and malaria disease. It can be speculated that in malaria disease, SCT reduces parasite densities and inhibit intracellular parasite growth, thereby, reducing the probability of suffering from severe malaria [46]. Additionally, molecular mechanisms through the upregulation of complex proteins have been proposed as protective against HIV-1 infection for individuals with SCT [44]. Similarly, a previous study in Western Kenya suggested that chronic malaria and elevated EBV viral loads are factors in eBL disease, rather than the presence of the SCT [14]. Therefore, these mechanisms cannot be definitively ascertained as protective in EBV susceptibility for individuals with SCT.

Worldwide, over 300 variants of the G6PD gene have been characterized [47]. The World Health Organization (WHO) has proposed the reclassification of G6PD based on the biochemical and phenotypic characteristics of variants [48, 49]. Genetic variations of the G6PD gene have been reported with significant clinical implications in different populations [15] as exemplified in West Africa and, within ethnically diverse populations in South Africa [50, 51]. The current study established the presence of non-synonymous mutations from the sampled population from Western Kenya (Mahidol 487G > A, Kaiping 1388G > A, Union 1360 C > T, and Viangchan 871G > A, Chinese4 392G > T). These mutations have been associated with known G6PD deficient phenotypes [52]. The G6PD Mahidol is associated with moderate enzyme activity, comparable to the African A- [53, 54], and is considered the predominant variant in Southeast Asia [55–57]. The G6PD Kaiping 1388G > A and Union 1360 C > T are the most common variants in Southern China [52]. The effects of the G6PD mutation in viral infections have previously been studied in Asia where the Mahidol variant is predominant and has no significant protective role against Dengue [58]. An ex vivo study from the same Asian population established that immune cells (monocytes) from G6PD patients were at a higher risk of viral infections [59] and recent studies have linked G6PD deficiency with susceptibility to coronavirus (COVID-19) [60, 61]. The current study established a high prevalence of these mutation types from the sampled population, but they did not influence EBV acquisition. In the West African and Asian populations, the G6PD 968 C and the Mediterranean 563 C > T variant were associated with enzyme deficiency and protection against severe malaria [51, 52]. The 968 C mutation has a similar phenotypic characteristic to the African A- variant [55]. It is estimated that within sub-Saharan Africa, 10% of the population have the phenotypic G6PD A (376 A > G) or G6PD A- (202G > A) mutation with the 376 A > G mutation as a modifier [62]. The current study did not establish a significant association between the African A or A- variants and EBV acquisition. It is however important to note that the distribution of G6PD variants within the malaria-endemic region of Western Kenya has solely been based on the detection of the A- haplotype [63], even though other studies have shown that the distribution of G6PD variants can vary based on geographical and ethnic groups [55, 57, 64, 65]. The current study highlights significant G6PD variants that have previously not been studied in this population for their role in EBV acquisition. Overall, the lack of protective association of the G6PD genotypes could suggest that the mechanism of protection of G6PD in malaria might differ from that in viral infections [58].

The EBV-associated eBL has a geographically similar distribution pattern to endemic malaria [20, 66]. In EBV-associated eBL pathogenesis, environmental, genetic, and immune-related factors have been implicated. For example, it has been established that children residing in malaria-endemic regions acquire EBV infection by six months of age on average, compared to those from non-endemic regions, thus implicating *P. falciparum* as well as EBV in breast milk as a causal factor in EBV transmission or eBL development [22, 67, 68]. Immune-related interactions in malaria and EBV infection share a disease-causal relationship considering that exposure to repeated malarial infections and early ages of EBV infections lead to eBL occurrence [24, 69, 70]. These facts have contributed to studies that aim to establish causal relationships between the two endemic diseases to inform interventions for eBL management [70].

A study of the MSP-2 genotype confirmed that antibodies against this blood-stage parasite gene are protective against severe malaria outcomes [71–73]. The current study aimed to establish the role of *in-utero* exposure to MSP-2 genotypes (3D7 and FC27) in the acquisition of EBV. Based on the fact that previous findings reported 35% of infants born in Western Kenya, compared to those from the highlands, are EBV-infected before 6 months of age [22]. This study explored how *in-utero* exposure to 3D7 and FC27 MSP-2 allelic variants alter susceptibility to EBV infection before and after 6 months of age. The current study did not find any relationship between *in-utero* exposure to MSP-2 and EBV acquisition. These findings are in line with previous observations in which there was no demonstration of a protective role of the gene in arthropod-borne viral infection [74]. The lack of an association of MSP-2 in EBV acquisition could therefore be attributed to immune-modulating mechanisms and the presence of other *P. falciparum* antigens that are not invoked in EBV infection [75–78]. Other mechanisms involving inhibition of erythrocyte invasion and replication, such as complement-dependent mechanisms [79], and elimination of infected blood cells by circulating immune cells in malaria disease, could also be factors [80]. Additionally, EBV could employ survival mechanisms that enable it to evade immune responses as has been established in its latency cycle within immune B-cells [81]. To the best of our knowledge, the current study established novel G6PD mutations within the population from western Kenya and suggests that further studies should explore enzyme levels and phenotypic characteristics for the G6PD variants detected in this population. Such future studies may offer important clinical relevance for the management and treatment of childhood malaria in endemic regions. Additionally, this study was limited in that larger sample sizes from multiple study sites and with higher age groups above 12 months of age would be required to demonstrate that SCT, α-thalassemia, G6PD, and MSP-2 mutations are not significantly associated with EBV acquisition. The current study only explored the value of a few genes that drive the differential presentation of malaria in EBV acquisition within one geographical region in Western Kenya. To establish that the known and novel hemoglobinopathies, and infant *in utero* MSP-2 exposure to EBV do not confer susceptibility to EBV, future studies with larger sample sizes and that use advanced techniques like transcriptome and epigenetics analysis could be used. This will highlight the gene variants that are important in EBV susceptibility.

## Conclusions

Based on the findings from this study, we conclude that SCT, α-thalassemia, G6PD mutations, and *in-utero* exposure to MSP-2 do not influence EBV susceptibility in infants from the malaria holoendemic region of Western Kenya. Therefore, hemoglobinopathies and *in-utero* exposure to MSP-2 genotypes do not appear to alter susceptibility to EBV acquisition.

## Electronic supplementary material

Below is the link to the electronic supplementary material.

Supplementary Material 1

## Data Availability

All data generated or analysed during this study are included in this published article [and its supplementary information files].

## List of abbreviations

DNA: Deoxyribonucleic Acid
eBL: Endemic Burkitt’s Lymphoma
EBV: Epstein Barr virus
G6PD: Glucose-6-Phosphate Dehydrogenase
MSP-2: Merozoite Surface Protein-2
NADPH: Nicotinamide Adenine Dinucleotide Phosphate
*P. f*: *Plasmodium falciparum*
PCR: Polymerase Chain Reaction
RBC: Red Blood Cell
SCT: Sickle Cell Trait
SCD: Sickle Cell Disease
µL: Microliter
α: alpha
HIV: Human Immunodeficiency Virus
NADPH: Nicotinamide Adenine Dinucleotide Phosphate
COVID-19: Corona Virus Disease-19
SNP Single: Nucleotide polymorphism
Hb AA: Sickle Cell Normal
